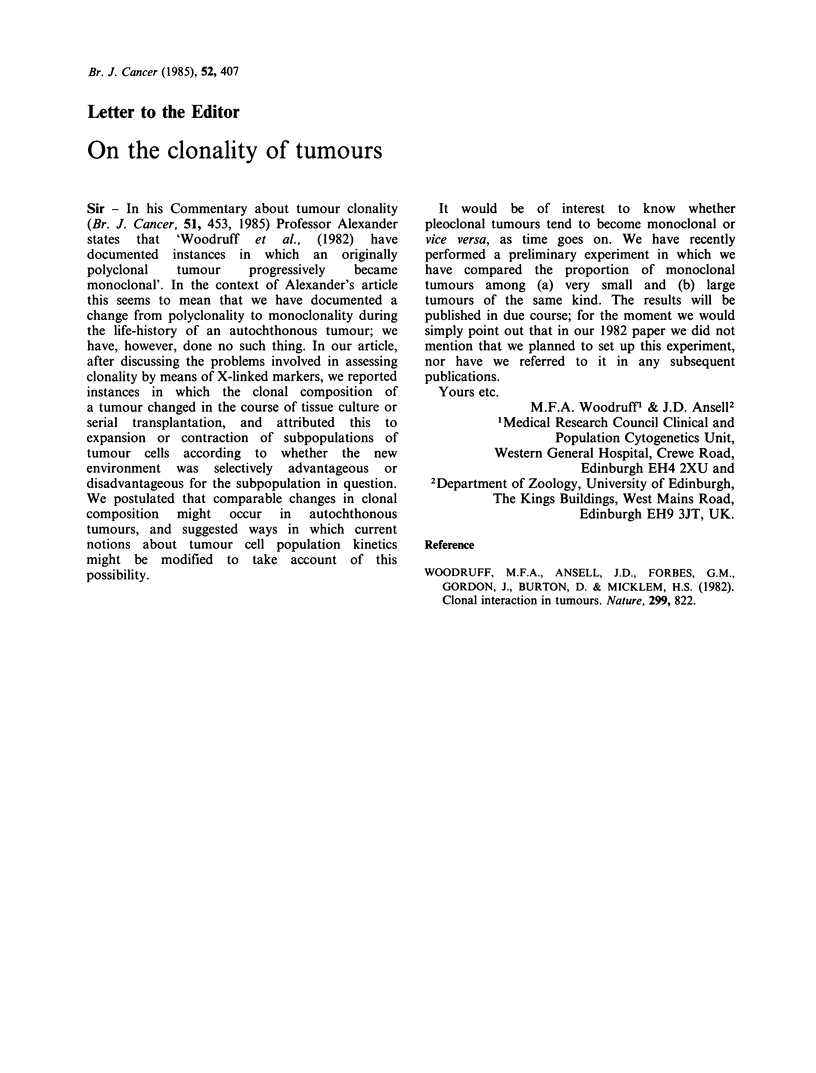# On the clonality of tumours.

**DOI:** 10.1038/bjc.1985.209

**Published:** 1985-09

**Authors:** M. F. Woodruff, J. D. Ansell


*Br. J. Cancer* (1985), **52**, 407

### Letter to the Editor

### On the clonality of tumours

**Sir** – In his Commentary about tumour clonality (*Br. J. Cancer,* **51,** 453, 1985) Professor Alexander states that 'Woodruff *et al.,* (1982) have documented instances in which an originally polyclonal tumour progressively became monoclonal'. In the context of Alexander's article this seems to mean that we have documented a change from polyclonality to monoclonality during the life-history of an autochthonous tumour; we have, however, done no such thing. In our article, after discussing the problems involved in assessing clonality by means of X-linked markers, we reported instances in which the clonal composition of a tumour changed in the course of tissue culture or serial transplantation, and attributed this to expansion or contraction of subpopulations of tumour cells according to whether the new environment was selectively advantageous or disadvantageous for the subpopulation in question. We postulated that comparable changes in clonal composition might occur in autochthonous tumours, and suggested ways in which current notions about tumour cell population kinetics might be modified to take account of this possibility.

It would be of interest to know whether pleoclonal tumours tend to become monoclonal or *vice versa*, as time goes on. We have recently performed a preliminary experiment in which we have compared the proportion of monoclonal tumours among (a) very small and (b) large tumours of the same kind. The results will be published in due course; for the moment we would simply point out that in our 1982 paper we did not mention that we planned to set up this experiment, nor have we referred to it in any subsequent publications.

Yours etc.

M.F.A. Woodruff[1] & J.D. Ansell[2]
[1]Medical Research Council Clinical and Population Cytogenetics Unit, Western General Hospital, Crewe Road, Edinburgh EH4 2XU and
[2]Department of Zoology, University of Edinburgh, The Kings Buildings, West Mains Road, Edinburgh EH9 3JT, UK.

### Reference

WOODRUFF, M.F.A., ANSELL, J.D., FORBES, G.M., GORDON, J., BURTON, D. & MICKLEM, H.S. (1982). Clonal interaction in tumours. *Nature,* **299,** 822.